# Biochemical Study of Oxidative Stress Markers in the Liver, Kidney and Heart of High Fat Diet Induced Obesity in Rats

**DOI:** 10.1186/1758-5996-3-17

**Published:** 2011-08-03

**Authors:** Saad A Noeman, Hala E Hamooda, Amal A Baalash

**Affiliations:** 1 Medical Biochemistry Department, Faculty of Medicine, Tanta University, Egypt

## Abstract

**Background:**

Obesity has become a leading global health problem owing to its strong association with a high incidence of diseases.

**Aim:**

To induce rat obesity using high fat diet (HFD) and to estimate oxidative stress markers in their liver, heart and kidney tissues in order to shed the light on the effect of obesity on these organs.

**Materials and methods:**

Sixty white albino rats weighing 150-200 g were randomly divided into two equal groups; group I: received high fat diet for 16 weeks, and group II (control group): received only normal diet (rat chow) for 16 weeks. Blood samples were taken for measurement of lipid profile, tissue samples from liver, heart and kidney were taken for determination of malondialdehyde (MDA), protein carbonyl (PCO), reduced glutathione (GSH) levels, and the activities of glutathione S- transferase (GST) glutathione peroxidase (GPx), catalase (CAT) and paraoxonase1 (PON1) enzymes.

**Results:**

Data showed that feeding HFD diet significantly increased final body weight and induced a state of dyslipideamia. Also our results showed a significant increase MDA and PCO levels in the hepatic, heart and renal tissues of obese rats, as well as a significant decrease in the activity of GST, GPx and PON 1 enzymes. On the other hand CAT enzyme activity showed significant decrease only in renal tissues of obese rats with non significant difference in hepatic and heart tissues. GSH levels showed significant decrease in both renal and hepatic tissues of obese animals and significant increase in their heart tissues. Correlation studies in obese animals showed a negative correlation between MDA and PCO tissue levels and the activities of GPx, GST and PON1 in all tissues and also with CAT enzyme activity in renal tissues. Also a negative correlation was detected between MDA & PCO tissues levels and GSH levels in both hepatic and renal tissues. While positive correlation was found between them and GSH levels in heart tissues.

**Conclusion:**

High fat diet-induced obesity is accompanied by increased hepatic, heart, and renal tissues oxidative stress, which is characterized by reduction in the antioxidant enzymes activities and glutathione levels, that correlate with the increase in MDA and PCO levels in most tissues. This may probably contribute to the additional progression of obesity related problems.

## Introduction

Obesity is a pathological condition in which excess body fat has accumulated to the extent that it may have an adverse effect on health, leading to reduced life expectancy and/or increased health problems [1]. The induction of obesity may be performed in animals by neuroendocrine, dietary or genetic changes [2]. The great similarity and homology between the genomes of rodents and humans make these animal models a major tool to study obesity [2].

Oxidative stress is highly correlated with a wide variety of inflammatory and metabolic disease states, including obesity [3, 4, and 5]. It is highly correlated with cumulative damage in the body done by free radicals inadequately neutralized by antioxidants [6]. It has been shown that free radicals may adversely affect cell survival because of membrane damage through the oxidative damage of lipid, protein and irreversible DNA modification [7]. Lipid peroxidation such as thiobarbituric acid reactive substances and hydroperoxides levels as well as markers of protein oxidation such as carbonyl proteins are markers of oxidative damage of ROS [8,9].

Furthermore oxidative damage is aggravated by the decrease in antioxidant enzymes activities such as superoxide dismutase, catalase (CAT), glutathione S-transferase (GST), and glutathione peroxidase (GPx) which acts as a free radical scavengers in conditions associated with oxidative stress [10].

Paraoxonase (PON1) is another antioxidant enzyme closely associated with high-density lipoproteins. It is a calcium-dependent esterase, which detoxifies lipid peroxides, and is widely distributed in many tissues, including the liver, brain, lung, heart, kidneys, small intestine and aorta [11].

Evidence suggests that a clustering of sources of oxidative stress exists in obesity; hyperglycemia, increased tissue lipid levels, inadequate antioxidant defenses, increased rates of free radical formation, and chronic inflammation [12]. Obesity affected many organs in the body such as liver, heart and kidney. Fatty liver and nephropathy are commons complication of obesity [13]. Arthrosclerosis and cardiac complications are more common among obese individuals [14,15].

Therefore the present study was designed to investigate the development of obesity in response to a high fat diet (HFD) and to estimate oxidative stress markers in the liver, heart and kidney in obese rats to shed the light on the effect of obesity on these organs.

## Materials and methods

This study was carried out on 60 white males and females' albino rats, their weight ranged between 150-200 g. During the study the animals were kept in wire mesh cages with ad libitum access to water. The room temperature was about 22-24°C and the animals were exposed to 12:12 hours light dark cycles. The animals were randomly divided into two equal groups:

Group I: received the high fat diet for 16 weeks.

Group II (control group): received only normal diet ad libitum (rat chow) for 16 weeks. Composition of the experimental diet (g/kg diet) was according to the formula of Kim et al. [16] with some modefications. It included the normal diet for control rats (Fat 5% [corn oil 5%], carbohydrates 65% [corn starch 15% and sucrose 50%], proteins 20.3% [casein 20% and DL-Methionine 3%], fiber 5%, salt mixture 3.7%, and vitamin mixture 1%). The high fat diet contained (fat 46% [corn oil 25.5%, and beef tallow 20.5%], carbohydrates 24% [corn starch 6% and sucrose 18%], proteins 20.3% [casein 20% and DL-Methionine 3%], fiber 5%, salt mixture 3.7%, and vitamin mixture 1%).

Normal and HFD constituents were purchased from El-Gomhoria Company, Cairo, Egypt. HFD was preserved at 4°C until used. Obesity was induced in 16 weeks.

Our work was carried out in accordance with the guidelines of the Ethical Committee of Medical Research of Faculty of Medicine Tanta University.

### Sample collection

By the end of the experimental period, all rats were sacrificed and blood samples were collected. Sera were separated and stored in aliquots at -70°C till used for estimation of lipid profile including; total cholesterol [17], triglycerides [18], LDL-cholesterol, VLDL [19], and HDL-cholesterol [20], by enzymatic colorimetric methods using commercial kits.

Then the abdomen and the thorax were opened and both liver, kidneys and heart were removed, washed three times in ice cold saline and blotted individually on ash-free filter paper, used for preparation of tissue homogenates for estimation of tissue MDA, PCO, GSH levels and the activity of GST, GPx, CAT and PON1 enzymes.

### Preparation of tissue homogenates

Specimens from each organ were separated into three parts. Each piece was weighted and homogenized separately with a potter- Elvenhjem tissue homogenizer *One part *was homogenized in phosphate buffer saline (PBS) 50 mM pH (7.4) for estimation of protein content, GST, CAT enzymes activities and GSH level, *the second *was homogenized in potassium phosphate buffer 10 mM pH (7.4) for estimation of MDA, PCO levels and GPx activity, and *the third *in Tris- HCl 100 mM, pH (8) for estimation of PON1 activity. The crude tissue homogenate was centrifuged at 10,000 rpm, for 15 minutes in cold centrifuge, and the resultant supernatant was used for the different estimations. Protein content in tissue homogenate was measured according to the method of Lowry et al. [21].

### Chemicals

Cummene hydroperoxide, 1-chloro-2, 4-dinitrobenzene (CDNB), 5-5-dithiobis-2-nitrobenzoic acid (DTNB) paraoxon and Dinitrophenylhydrazine (DNPH) were obtained from (Sigma chemical Co. St., Louis, MO, USA). Thiobarbituric acid (TBA) and reduced glutathione (GSH) were obtained from Fluka Chemical Co. Trichloroacetic acid (TCA) were obtained from Merk Chemical Co USA. Acetic acid, potassium dichromate, hydrogen peroxide, hydrochloric acid and ethanol were obtained from El-Nasr chemical Co.

### Spectrophotometric determination of tissues MDA, PCO, and GSH levels

#### MDA

This method depends on the formation of MDA as an end product of lipid peroxidation which reacts with thiobarbituric acid producing thiobarbituric acid reactive substance (TBARS), a pink chromogen, which can be measured spectrophotometrically at 532 nm, an MDA standard was used to construct a standard curve against which readings of the samples were plotted [22].

#### PCO

This method depends on the formation of a Schiff base from the reaction of dinitrophenylhydrazine with protein carbonyls to form protein hydrazones which was measured spectrophotometrically. Briefly, after precipitation of protein with an equal volume of 1% trichloroacetic acid, the pellet was resuspended in 10 mmol/L DNPH plus 2N HCl, or in 2N HCl as a control blank. Next, after the washing procedure with 1:1 ethanol-ethylacetate, the final palette was dissolved in 6 mol/L guanidine. The carbonyl group was determined from the absorbance at 370 nm. The carbonyl content was calculated in terms of nmol/mg protein [23,24].

#### GSH

The method is based on the reduction of 5,5 dithiobis (2-nitrobenzoic acid) (DTNB) with reduced glutathione (GSH) to produce a yellow compound. The reduced chromogen is directly proportional to GSH concentration and its absorbance can be measured at 405 nm by using a commercial kit was used (Biodiagnostic, Egypt) [25].

### Enzymatic Assays

*Tissues GST enzyme activity: *it measures the conjugation of 1-chloro-2,4-dinitro benzene (CDNB) with reduced glutathione that produces a dinitrophenyl thioether which can be detected by spectrophotometer at 340 nm. One unit of GST activity is defined as the amount of enzyme producing 1 mmol of CDNB-GSH conjugate/min under the conditions of the assay according to the method described by Habig et al. [26].

*Tissues GPx enzyme activity: *it was measured as IU/gm wet tissue by the reaction between glutathione remaining after the action of GPx and 5, 5-dithiobis-(2-nitrobenzoic acid) to form a complex that absorbs maximally at 412 nm. Glutathione peroxidase activity of 1 U/mg protein was defined as 1 μg of GSH consumed/min/mg protein [27].

*Determination of tissues CAT enzyme activity: *it assayed by the method of Sinha which based on formation of chromic acetate from dichromate and glacial acetic acid in presence hydrogen peroxide, chromic acetate that produced was measures colorimetrically at 570 nm, one enzyme unit was defined as the amount of enzyme which catalyzed the oxidation of 1 μmole H_2_O_2 _per minute under assay conditions [28].

*Tissues PON1 enzyme activity: *PON1 activity towards paraoxon (O,O-diethyl-O-p-nitrophenyl phosphate) was determined by measuring the initial rate of substrate hydrolysis to p- nitrophenol, whose absorbance was monitored at 405 nm in the assay mixture, A PON1 activity of 1 U/mg protein was defined as 1 μmol p-nitrophenol formed per minute per mg protein [29].

### Statistical analysis

All the statistical analyses were processed using Statistical Program of Social Sciences (SPSS) for windows, version 10.0. Values of the measured parameters were expressed as mean value ± SD and the difference between the two groups was determined using unpaired student's t-test, and the significance was considered at p values <0.05. Correlation between variables was evaluated using Pearson's correlation coefficient with level of significance <0.05.

## Results

**Figure 1:**
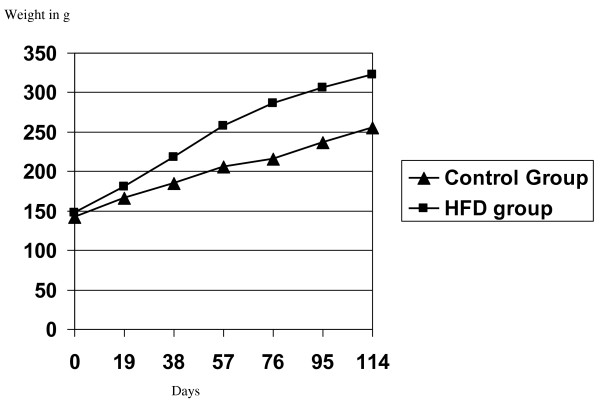
**Comparison between the two studied groups as regards body weight**.

By the end of the 16 weeks there was a significant increase in the weight of high fat diet fed rats; (group I) compared to the control (group II), (Figure 1). Also there was significant increase in serum levels of total triglycerides, total cholesterol, LDL-cholesterol and VLDL-cholesterol with low level of HDL-cholesterol in obese rats as compared with the control (Table 1).

**Table 1:** Statistical comparison between all studied groups as regards to lipid profile

Parameter	Group I (n = 30)	Group II (n = 30)	P
**Total triglycerdies (mmol/L)**	0.92 ± 7.75	0.64 ± 9.19	P <0.05*
**Total cholesterol (mmol/L)**	3.27 ± 0.23	2.32 ± 0.05	P <0.05*
**LDL-cholesterol (mmol/L)**	1.74 ± 0.043	0.86 ± 0.05	P <0.001*
**HDL-cholesterol (mmol/L)**	1.02 ± 0.06	1.9 ± 0.05	P <0.001*
**VLDL-cholesterol (mmol/L)**	0.79 ± 0.01	0.36 ± 0.01	P <0.001*

Our results showed a statistically significant increase in the hepatic tissues levels of MDA and PCO in obese rats when compared to the control group, (p < 0.05). Concerning the antioxidant enzymes activities in obese animals, the present study showed a statistical significant decrease in the activities of hepatic GST, GPx and PON 1 enzymes, and also decrease in hepatic GSH level in obese rats compared to control. Non significant difference was found in CAT enzyme activity between the two groups, (p >0.05) (Table 2).

**Table 2:** Statistical comparison between all studied parameters in liver tissues.

Parameter	Group I (n = 30)	Group II (n = 30)	P
**MDA (nmol/g wet tissue)**	93.30 ± 9.63	21.7 ± 5.66	P <0.05*
**Protein carbonyl (nmol/g wet tissue)**	26.44 ± 2.32	20.16 ± 1.13	P <0.05*
**GSH (mg/g/g wet tissue)**	93.32 ± 9.62	124.2 4 ± 1.39	P <0.05*
**GST (μmol/g wet tissue)**	343.7 ± 21.36	470.9 ± 22.9	P <0.05*
**GPx (IU/g wet tissue)**	21.85 ± 2.68	29.14 ± 1.76	P <0.05*
**CAT (μmol H2O2 consumed/mg protein/min)**	664.26 ± 6.66	665.80 ± 6.77	P > 0.05
**PON 1 (U/mg protein/min)**	151.833 ± 61.55	284.31 ± 110.60	P <0.05*

The present study also showed significant increase heart tissues level of MDA and PCO in obese animals than the control. There was significant decrease in heart GST, GPx, and PON 1 enzymes activities, with no significant difference in CAT enzyme activity in obese rats, (p >0.05). GSH heart tissues level showed a significant increase in obese animals (Table 3).

**Table 3:** Statistical comparison between all studied parameters in the heart tissues.

*Parameter*	*Group I* *(n = 30)*	*Group II* *(n = 30)*	P
**MDA (nmol/g wet tissue)**	29.38 ± 4.75	48. 99 ± 4.40	P <0.05*
**Protein carbonyl (nmol/g wet tissue)**	18.4 ± 1.95	22.86 ± 2.95	P <0.05*
**GSH (mg/g/g wet tissue)**	90.54 ± 5.59	98.2 ± 3.67	P <0.05*
**GST (μmol/g wet tissue)**	289.6 ± 16.95	261.158 ± 62.09	P <0.05*
**GPx (IU/g wet tissue)**	26.1 ± 2.06	20.9 ± 2.06	P <0.05*
**CAT (μmol H2O2 consumed/mg protein/min)**	591.8 ± 4.75	586.5 ± 9.5	P >0.05
**PON 1 (U/mg protein/min)**	188.54 ± 58.62	115.74 ± 30.26	P <0.05*

Table 4 showed significant increase of renal tissue levels of MDA and PCO in obese rats as compared to the control group. Also there was a significant decrease in the activities of renal GST, GPx, CAT and PON 1 enzymes in obese rats. GSH level showed a significant decrease in obese rats than the control.

**Table 4:** Statistical comparison between all studied parameters in the kidney tissues.

*Parameter*	*Group I* *(n = 30)*	*Group II* *(n = 30)*	P
**MDA (nmol/g wet tissue)**	69.09 ± 6.43	41.32 ± 4.79	P < 0.001*
**Protein carbonyl (nmol/g wet tissue)**	26.54 ± 3.4	18.86 ± 1.69	P < 0.001*
**GSH (mg/g wet tissue)**	89.89 ± 1.9	101 ± 2.38	P < 0.001*
**GST (μmol/g wet tissue)**	223.97 ± 10.459	258.76 ± 16.6	P < 0.001*
**GPx (IU/g wet tissue)**	20.94 ± 2.85	28.55 ± 4.779	P < 0.001*
**CAT (μmol H2O2 consumed/mg/protein/min)**	577.37 ± 7.69	585.9 ± 3.7	P < 0.001*
**PON 1 (U/mg protein/min)**	93.23 ± 12.45	112.43 ± 15.32	P < 0.001*

The correlation matrix in our study showed that in obese animals there was a negative correlation between MDA and PCO levels and the activities of GPx, GST and PON1 in the hepatic, heart and renal tissues and with CAT enzyme activity in renal tissues. No correlation was detected between MDA or PCO tissue levels and CAT enzyme activity in the hepatic and heart tissues. A negative correlation was detected between MDA, PCO levels and GSH levels in both hepatic and renal tissues. Positive correlation was found between heart MDA and PCO levels and the GSH level (Tables 5, 6, 7).

**Table 5:** Correlation matrix between MDA, PCO levels and the studied antioxidants in liver tissues.

		*GSH*	*GST*	*GPx*	*CAT*	*PON1*
**MDA**	r	-0.642	-0.423	-0.796	-0.325	-0.652
	P-value	0.002	0.008*	0.001*	0.475	0.025*

**PCO**	r	0.410	-0.685	-0.586	0.319	-0.420
	P-value	0.001*	0.001*	0.017*	0.112	0.039*

**Table 6:** Correlation matrix between MDA, PCO levels and the studied antioxidants in heart tissues.

		*GSH*	*GST*	*GPx*	*CAT*	*PON1*
**MDA**	r	0.597	-0.841	-0.684	-0.201	-0.583
	P-value	0.042*	0.041*	0.021*	0.523	0.030*

**PCO**	r	0.410	0.685	0.586	0.355	-0.523
	P-value	0.001*	0.001*	0.017*	0.099	0.022*

**Table 7:** Correlation matrix between MDA, PCO levels and the studied antioxidants in kidney tissues.

		*GSH*	*GST*	*GPx*	*CAT*	*PON1*
**MDA**	r	-0.538	-0.417	-0.745	-0.492	-0.639
	P-value	0.022*	0.028*	0.044*	0.020*	0.011*

**PCO**	r	0.410	0.685	0.586	0.458	-0.752
	P-value	0.001*	0.001*	0.017*	0.007*	0.009*

## Discussion

Obesity is a strong risk factor for developing dyslipidemia [30,31], diabetes mellitus [32], fatty liver (which can later progress to nonalcoholic fatty liver disease [33], cardiovascular (CV) diseases such as heart failure (HF) and coronary heart disease (CHD) [15].

Feeding of (HFD) to rats was proved to be a useful model of putative effects of dietary fat in humans [34]. Rat models are therefore useful tools for inducing obesity as they will readily gain weight when fed high-fat diets [2].

In the present study, obesity was induced in white albino rats by using a high fat diet formula. Obesity was induced in 16 weeks. The weight gained by rats fed HFD formula, was significantly more than that gained by those fed the normal diet. Many workers were able to induce obesity in rats using different formulas of high fat diets [16,35-38]. The response of animals to the HFD is a subtle but cumulative effect, because it took over a 10 weeks period. The difference in weight gain in all above studies may be due to age, genetic makeup of the different strains and composition of different formulas [35,36].

HDF resulted in dyslipidemic changes as illustrated by increasing serum levels of triacylglyceral, total cholesterol, LDL-cholesterol and VLDL-cholesterol and low level of HDL cholesterol as compared with control; a finding in accordance with that of Woo et al [39], and Kamal and mohamed [40]. Dyslipidemic changes occurs in obesity may be due to the increased triacylglycerol, content of the liver due to increased influx of excess NEFAs into the liver[41]. It has been revealed that altered lipid concentrations and qualitative changes of the lipoprotein fractions in obesity are associated with an increased risk of various adverse effects of obesity [42,43]. Additionally, lipid alterations have been considered as contributory factors to oxidative stress in obesity [44]. Increased production of reactive oxygen species as well as reduced antioxidant defense mechanisms have been suggested to play a role in both humans and animal models of obesity [3,45].

Lipid peroxidation is thought to be a component of obesity-induced pathology [46]. The data presented in this study showed that obesity increased lipid peroxidation in hepatic, cardiac and renal tissues as expressed by increased tissue levels of MDA.

Our results are in basic agreement with the results of Vincent, et al., [47], Olusi et al., [8], and Amirkhizi et al., [46] who showed that, obesity is an independent risk factor for increasing lipid peroxidation and decreased activity of cytoprotective enzymes. Obesity can cause increased lipid peroxidation by progressive and cumulative cell injury resulting from pressure of the large body mass. Cell injury causes the release of cytokines, especially tumor necrosis factor alpha (TNF-α) which generates ROS from the tissues which in turn cause lipid peroxidation [48]. The hypertriglyceridemia seen in obese rats may contribute to the alteration in the oxidant-antioxidant balance, suggesting that an increase in the bioavailability of free fatty acids can increase lipid peroxidation [46].

Cellular proteins are believed to be the target of free radical-induced oxidative injury. Protein carbonyl (PCO) content of liver, heart and kidney is increased significantly in obese rats compared to normal rats. Increasing PCO in obesity may be due to damage of cellular proteins by ROS generated in obesity. The accumulation of oxidized proteins might impair the cell function. The use of PCO as a marker for measuring of damaged proteins may have some advantages over other markers, because of relatively early formation, greater stability and reliability and also their longer life-span [49].

There are several potential mechanisms for of the increasing lipid peroxidation and protein carbonyl in hepatic, cardiac and renal tissues. Reactive oxygen species (ROS) and lipid peroxidation products impaired the respiratory chain in hepatocytes either directly or indirectly through oxidative damage to the mitochondrial genome. These features, in turn, lead to the generation of more ROS, and a vicious cycle ensues. Mitochondrial dysfunction can also lead to apoptosis or necrosis depending on the energy status of the cell. Finally, ROS and lipid peroxidation products also activate stellate cells, thus resulting in fibrosis [50,51].

Lipid peroxidation in the heart leads to loss of the cellular membrane integrity due to oxidative modification of lipids and proteins that can ultimately lead to cardiac arrhythmias, poor contractility, infarction, cardiac failure or sudden death [47]. The potential mechanism for increased lipid peroxidation in cardiac tissue may be due increased lipid substrate within the myocardium in which can serve as a larger target for oxidation by free radicals [47,52]. It is well established that elevated myocardial work and mechanical overload is associated with increased free radical production consequently lipid peroxidation [53]. Mechanical overload-induced increases in muscle oxygen consumption accelerate electron flux through the mitochondria in proportion to the need for ATP. This results in increased electron leakage from the electron transport chain and increased production of superoxide anions [47].

Several mechanisms may contribute to the onset and/or the progression of renal involvement in experimental obesity among them; lipid peroxidation and oxidative stress have been frequently proposed. HFD induces alteration of renal lipid metabolism by an imbalance between lipogenesis and lipolysis in the kidney, as well as systemic metabolic abnormalities and subsequent renal lipid accumulation and lipid peroxidation leading to renal injury [54]. The accumulation of adipose tissue around the kidneys of obese rats penetrates into the medullary sinuses thus increased intrarenal pressures which may cause damage the renal tissue. Damaged renal tissue acts as sources of ROS and develops lipid peroxidation. An increased lipid peroxidation in the kidney tissue, as well as modification of the circulating LDL/VLDL fraction, is probably involved in the onset of kidney lesions in this normoglycaemic rodent model of obesity [55].

It has been shown that animal body had an effective mechanism to prevent the free radical induced tissue cell damage, this accomplished by a set of endogenous antioxidant enzymes and protein such as GST, SOD, CAT, GPX, GRD and GSH. When the balance between ROS production and antioxidant defense is lost oxidative stress results; which through a serious of events deregulates the cellular functions leading various pathological conditions [10]. GST, CAT and GPX constituted a mutually supportive team of defense against reactive oxygen species. In the present study GST, CAT, GPX and PON 1 enzymes activity and GSH protein were measured in hepatic, cardiac and renal tissue and the data showed clearly a significant decrease in the activities of GST, GPx and PON 1 enzymes in liver, heart and renal tissues in obese rats as compared to the control group. Catalase (CAT) enzyme showed a non significant change in hepatic and heart tissues and decreased in renal tissues of obese rats. GSH level showed significant decrease in both liver and renal tissues in obese rats. While its level in heart tissues showed significant increase in obese rats. Our results were in agreement with many authors [[8,47,50] and [55]]. There are several mechanisms explaining the reduction of antioxidant enzymes in obese rats;

The increased lipid peroxidation lead to inactivation of the enzymes by crosses linking with MDA; this will cause an increased accumulation of superoxide, H_2_O_2 _and hydroxyl radicals which could further stimulate lipid peroxidation. This mechanism has a clue from work of Demori et al., (2006) [56] and Moyà et al., 2008 [50] who showed that the liver catalase, glutathione peroxidase, and Mn-superoxide dismutase were reduced in response to the cafeteria-diet feeding in obese rats. Furthermore our correlation study indicated that there is negative correlation between MDA and PCO levels and enzymes activities of GPx, GST and PON 1 in the liver, heart and renal tissues and with CAT enzyme activity in kidney. This correlation finding conformed and supported the concept of inactivation of antioxidant enzymes and proteins by high level of lipid peroxidation in obesity.

Decrease of antioxidant enzyme may be due to rapid consumption and exhaustion of storage of this enzyme in fighting free radicals generated during development of obesity.

PON-1 activity could be decreased as consequence of an altered synthesis and/or secretion of HDL secondary to impaired lecithin cholesterol acyl transferase (LCAT) activity.

Under oxidative stress, PON-1 may be inactivated by Sglutathionylation a redox regulatory mechanism characterized by the formation of mixed disulfide between a protein thiol and oxidized glutathione [57].

In the present study, the non significant change in the CAT enzyme activity in liver and heart tissues of obese rat may be due to increases oxygen consumption of previous tissues due to obesity and the change of CAT enzyme activity is dependent on oxygen consumption [12,47]. As stated above, cardiac tissue of obese rats showed significant high content of GSH compared to normal rats. Vincent1, et al., demonstrated similar results. Increasing of GSH concentration in cardiac tissue in obese rats in response to free radical formation in an effort to protect cells against oxidative damage [47]. The adaptation of the primary antioxidant defense in the hearts of high-fat-fed animals appeared to be less complete, as indicated by the failure of other antioxidant enzyme activities including GST, GPX and PON 1 to increase in the hearts of animals on the high-fat diet. Non significant correlation was detected between MDA or PCO levels and CAT enzyme activity in the liver and heart tissues.

## Conclusion

High fat diet-induced obesity is accompanied by increased hepatic, heart, and renal tissues oxidative stress, which is characterized by reduction in the antioxidant enzymes activities and glutathione levels, that correlate with the increase in MDA and PCO levels in most tissues. This may probably contribute to the additional progression of obesity related problems.

## Competing interests

The authors declare that they have no competing interests.

## Authors' contributions

HEH and AAB participated in animal preparation, sample collection, and biochemical assays, and together with SAN participated in the design of the study, performed statistical analysis, and drafted the manuscript. All authors read and approved the final manuscript.
